# Experimental system for measurement of radiologists’ performance by visual search task

**DOI:** 10.1186/2193-1801-2-607

**Published:** 2013-11-14

**Authors:** Eriko Maeda, Takeharu Yoshikawa, Ryoichi Nakashima, Kazufumi Kobayashi, Kazuhiko Yokosawa, Naoto Hayashi, Yoshitaka Masutani, Naoki Yoshioka, Masaaki Akahane, Kuni Ohtomo

**Affiliations:** Department of Radiology, Graduate School of Medicine, University of Tokyo, 7-3-1 Hongo, Bunkyo-ku, Tokyo, 113-8655 Japan; Department of Psychology, Graduate School of Humanities and Society, University of Tokyo, 7-3-1 Hongo, Bunkyo-ku, Tokyo, 113-8655 Japan; Department of Radiology, Sanno Hospital, International University of Health and Welfare, 8-10-16, Akasaka, Minato-ku, Tokyo, 107-0052, 107-0052 Japan

**Keywords:** Radiologist, Performance, Visual search task, Receiver operating characteristic analysis, Reaction time

## Abstract

**Purpose:**

Detective performance of radiologists for “obvious” targets should be evaluated by visual search task instead of ROC analysis, but visual task have not been applied to radiology studies. The aim of this study was to set up an environment that allows visual search task in radiology, to evaluate its feasibility, and to preliminarily investigate the effect of career on the performance.

**Materials and methods:**

In a darkroom, ten radiologists were asked to answer the type of lesion by pressing buttons, when images without lesions, with bulla, ground-glass nodule, and solid nodule were randomly presented on a display. Differences in accuracy and reaction times depending on board certification were investigated.

**Results:**

The visual search task was successfully and feasibly performed. Radiologists were found to have high sensitivity, specificity, positive predictive values and negative predictive values in non-board and board groups. Reaction time was under 1 second for all target types in both groups. Board radiologists were significantly faster in answering for bulla, but there were no significant differences for other targets and values.

**Conclusion:**

We developed an experimental system that allows visual search experiment in radiology. Reaction time for detection of bulla was shortened with experience.

## Introduction

Radiologists have been interested in measuring their performance to know the effect of factors such as modality, reconstruction method, MR sequence, or experience on reading. After the introduction of receiver operating characteristic (ROC) analysis into the field of radiology in 1971 by Lusted, radiologists have been almost exclusively using ROC analysis for studies comparing radiologists’ performance under different conditions (Lusted 1971). The merit of ROC analysis is that sensitivity and specificity can be known for any cut-off value, and also that the best cut-off value can be determined from ROC curve (Obuchowski 2003; Metz 1978). By comparing ROC curves in different conditions, we can also know the best condition by finding the curve closest to the left upper corner. The key of ROC analysis in radiology is that the participants rate the confidence of judgment or the likelihood of malignancy etc. instead of giving binary answer (i.e. present or absent) (Obuchowski 2003; Metz 1978; Hanley & McNeil 1982; Berbaum et al. 1989; Metz 1989; Gur et al. 1989). The fundamental problem of rating is that the decision needs to be “not obvious”, and “should be of borderline difficulty” (Metz 1978). This means ROC analysis needs careful selection of images, and is not suitable when the searched target is obvious, which is often the case in practice. From another point of view, every radiologist is making effort to avoid overlooking errors, but they sometimes happen, even for obvious targets. To avoid simple error of overlooking obvious targets, a variety of computer assisted detection (CAD) programs has been developed. CAD programs detect candidates of lesions such as lung nodules and cerebral artery aneurysms. These lesions may be of various conspicuity, but once found, they are usually obvious and radiologists usually answer “confident” when their level of confidence for the lesion presence is asked. In such case, ROC analysis is not suitable for evaluation of performances of radiologists and CADs, because the decision is binary. We need a new method to evaluate simple detection performance in radiology.

Although research of image perception is minor in radiology, it has been studied as the main target of research in the field of cognitive psychology, using “visual search task” as well as ROC analysis (Kundel 2006; Wolfe 2010). In visual search tasks, background images with distractors are presented, certain percentage of them with a target image. Participants are asked to answer the presence or absence of a target among a set of distractors, typically by simply pressing a button. Examples of famous tasks of this kind are found in horizontal line search among vertical line distractors, letter “L” search among “T”s, and artificial baggage-screening task searching for “tools” among objects from other categories (Treisman & Gelade 1980; Wolfe et al. 2005; Rubinstein 2001; Schwaninger et al. 2005). Results are typically obtained in the forms of accuracy and reaction times. Efficacy of visual search task is influenced by number and feature of distractors. If the target has only one different feature from the distractors, such as color, size, direction, and shape, the task is easy, and rapid. The example of this task is finding a red O or green X from numerous Os. The task becomes difficult when the participant is searching for a target that has a combination of more than one different feature from the distractors, for example finding a red P from a mixture of black Ps and red Bs. Based on these results, “feature integration theory” was proposed, and it is thought to be due to early processing for one feature is independent of other features (Treisman & Gelade 1980). The advantages of visual search tasks are unnecessity of rating, feasibility of using obvious targets, simplicity of image preparation, and available accuracy and reaction time figures in controlled environments. The method has the disadvantages of being empirical and time-consuming.

For the studies of optical cognitive functions, we need to be aware of the fact that there are situations suitable for visual search tasks but not ROC analysis, or the other way around. When applying the fact to medical images, research on detective performance of high-contrast lesions such as lung nodules on CT, associated with less optical ambivalence, is by nature suitable for visual search tasks rather than ROC analysis. However in radiology, ROC analyses have been applied to situations that are suitable for visual search. In one reason, this is because the visual search experiment is not feasible in ordinary radiology reading room, and requires preparation of empirical images and the system controlling their presentation or measuring precise reaction time (Nakashima et al. 2013; Nomura et al. 2010). There have been no past reports on evaluation of radiologists’ perceptive performance using visual search task. The aim of this study was to set up an environment that allows visual search task in radiology and to evaluate its feasibility. The other aim was to investigate the effect of career on detective performance using that system.

## Materials and methods

### Participants

Ten healthy radiologists (age 26–41 years; 2–16 years of experience in radiology; 9 males and 1 female) participated in the experiment. There were 4 radiologists with board certification by Japanese Radiological Society (age 32–41; 8–16 years of experience in radiology), and 6 radiologists without (age 26–31; 2–7 years of experience). All participants had normal or corrected-to-normal vision. The experiment was approved by the institutional review board and written informed consent was obtained from all participating radiologists. Written informed consent was waived for patients whose CT images of ground-glass nodules and solid nodules were processed and used for this experiment, because it was anonymous and retrospective use of cut-out lesions.

### Stimuli preparation

The whole part of what is presented to the radiologists on the display is called “stimuli” in visual search task. 250 CT slices of healthy lungs without any findings (20.7 × 20.7 cm) were prepared from screening examinations. Each slice was used eight times to prepare 2000 background CT images.

**Figure 1:**
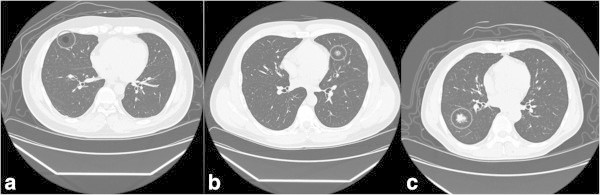
**Three types of targets.** Lesions within the gray circles are targets; **a** bulla, **b** ground-glass nodule (GGN) and **c** solid nodule (SN). Lesions were not indicated with gray circles in the experiment.

Three types of target lesion images were prepared: bulla without wall, pure ground-glass nodule (GGN) and solid nodule (SN). To create a target-present image, one lesion was inserted onto one of the background images. For GGN and SN, the image cut-out from the clinical case of primary lung carcinoma, reduced in size, rotated or inverted to make variations, were used. For bulla, black circles or ovals drawn on transparent background using Adobe Photoshop CS version 8.0.1 (Adobe Systems, San Jose, CA, USA) were used. Images of bulla cut-out from the clinical CT were not used because they were unnaturally conspicuous when inserted on background CT. Thus 24 patterns of bulla, 16 patterns of GGN and 8 patterns of SN were prepared (Figure 1a-c). Sizes of the targets were 8 × 8 mm for bulla and GGN and 10 × 10 mm for SN: SN had to be larger than the other two because SN had to be discerned from grouped blood vessels on one slice. The brightness and the contrast of each cut-out was adjusted to each background to avoid standing out. When inserting the target to the background, each lung field was divided into octant, and random digit list was used to allocate each pattern of targets on each octant with a constant probability. Within the octant, target positions were carefully allocated to avoid anatomical inconsistency, yet preventing spatial biases. A board radiologist who did not participate in the experiments supervised the whole image preparation.

### Stimuli presentation

Presentation of stimuli and response recording were controlled by Matlab software (MathWorks, Natick, MA, USA), using the Psychophysics Toolbox extensions installed on a laptop computer (HP Compaq tc4400 Tablet PC, Hewlett-Packard, Palo Alto, CA, USA) (Brainard 1997; Pelli 1997). Stimuli were displayed on a 22-inch monitor (1024 × 768 pixels; Diamondtron Flat RDF22H, Mitsubishi Electric, Tokyo, Japan). Participants viewed the monitor from a distance of 70 cm (16.5° × 16.5° of visual angle, fixed by a chin rest) in a dark room. This size is almost equal to the system used in traditional visual search tasks (Wolfe et al. 2005; Wolfe et al. 2007; Fleck & Mitroff 2007).

### Procedure

**Figure 2:**
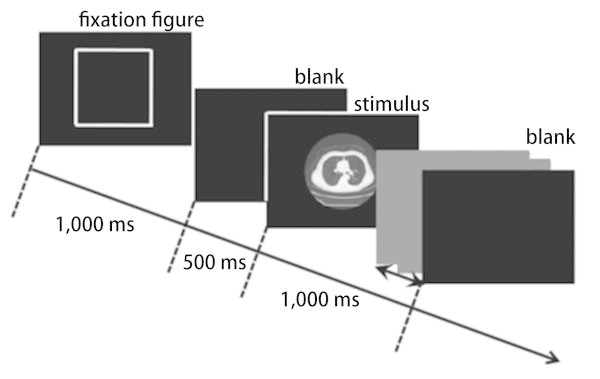
**On each trial, a fixation figure was presented for 1,000 ms, followed by a blank display presented for 500 ms and the stimulus.** The stimulus was presented until participants responded or after 1000 ms.

The 2000 images were divided into 8 sessions of 250 equivalent trials (each including 125 target-absent trials, 100 bulla-presented trials, 20 GGN-presented trials and 5 SN-presented trials). Participants had to complete all session at one sitting experiment, but were allowed to take free breaks between sessions. On each trial, a fixation figure (a hollow square of the same size as the stimuli) was presented for 1,000 ms, followed by a blank display presented for 500 ms and the stimulus. The stimulus was presented until participants responded or after 1,000 ms: the time limit set to prompt fast response. Participants were asked to respond as fast and accurately as possible by pressing a button on a numerical keypad (NT-USB19EC, Sanwa-supply, Okayama, Japan). Regarding the keypad, one number was allocated for one type of target (“00” for target-absent image, “1” for bulla, “2” for GGN, and “3” for SN). After the participants’ response, the next trial began after 500 ms presentation of a blank display (Figure 2). Participants had to respond even when they could not discern the presence or the type of the target because the onset of next trial was contingent on participant’s response. The time from the start of presentation of the stimulus until the response was recorded as the reaction time.

### Statistical analysis

Data from the trials with the reaction time of longer than 4,000 ms were excluded, because the participants were unlikely to be responsible for that answer. Long reaction time was associated with unavoidable incidence such as being very sleepy, getting called, or attention distracted by an earthquake.

True (i.e. correct) response was defined as a response indicating correct target type for each trial. For each trial, the data of true-false of the response (in the form of 1 or 0) and the reaction time were recorded. Although blinded to the participant, the stimuli were numbered from 1 to 2000 and the response and the reaction time for each stimulus could be sorted by stimulus number.

For statistical analysis, the participants were divided into two groups depending on board certification. For each stimulus number, the average true-false response (i.e. the accuracy) and the reaction time were calculated for the two groups. Between the two groups, accuracy was compared with Wilcoxon signed-rank test, and reaction time was compared with Student’s t-test. Sensitivity, specificity, positive predictive values and negative predictive values for each type of target (bulla, GGN, SN) were calculated for each participant. The averages of these values were also compared between the two groups using Student’s t-test.

Statistical significance was set at p < 0.05. After Bonferroni correction, statistical significance was defined as p < 0.0125.

## Results

All ten participants completed the experiment. Of the 20,000 trials of 10 participants, 24 trials of 9 participants were excluded because the reaction time exceeded 4,000 ms.

Accuracy of the participants for each types of target did not have any significant difference depending on board certification (Table 1). For reaction time, board participants were significantly faster in reacting for bulla targets, but there were no significant difference for other targets (Table 2).

**Table 1:** **Accuracy of the participants for each types of target**

	Non-board	Board	p value
Without target	0.99 ± 0.02	0.99 ± 0.03	0.92
Bulla	0.96 ± 0.11	0.95 ± 0.10	0.22
Ground-glass nodule	0.98 ± 0.05	0.98 ± 0.07	0.83
Solid nodule	0.98 ± 0.05	0.98 ± 0.07	0.44

**Table 2:** **Reaction time of the participants for each types of target**

	Non-board (sec)	Board (sec)	p value
Without target	0.84 ± 0.10	0.86 ± 0.15	0.99
Bulla	0.72 ± 0.10	0.71 ± 0.10	0.0024*
Ground-glass nodule	0.86 ± 0.15	0.86 ± 0.12	0.48
Solid nodule	0.90 ± 0.07	0.89 ± 0.08	0.29

*Statistical significance.

No significant difference was found for sensitivity, specificity, positive predictive values and negative predictive values for each type of target between board participants and non-board participants (Tables 3, 4, 5 and 6).

**Table 3:** **Sensitivity of the participants for each types of target**

	Non-board	Board	p value
Bulla	0.959 ± 0.024	0.954 ± 0.030	0.61
Ground-glass nodule	0.983 ± 0.011	0.978 ± 0.018	0.68
Solid nodule	0.983 ± 0.020	0.981 ± 0.023	0.89

**Table 4:** **Specificity of the participants for each types of target**

	Non-board	Board	p value
Bulla	0.997 ± 0.001	0.995 ± 0.003	0.88
Ground-glass nodule	0.9995 ± 0.0005	0.999 ± 0.0	0.11
Solid nodule	0.9997 ± 0.0005	0.9995 ± 0.0006	0.32

**Table 5:** **Positive predictive value of the participants for each types of target**

	Non-board	Board	p value
Bulla	0.989 ± 0.014	0.992 ± 0.005	0.31
Ground-glass nodule	0.995 ± 0.006	0.993 ± 0.0005	0.63
Solid nodule	0.992 ± 0.013	0.982 ± 0.023	0.40

**Table 6:** **Negative predictive value of the participants for each types of target**

	Non-board	Board	p value
Bulla	0.974 ± 0.014	0.970 ± 0.019	0.62
Ground-glass nodule	0.999 ± 0.0008	0.998 ± 0.002	0.73
Solid nodule	0.9995 ± 0.0005	0.9995 ± 0.0006	1.0

## Discussion

This is the first study to introduce a system that enables visual search task in radiology, and quantification of detective performance of radiologists in terms of accuracy, sensitivity, specificity, positive and negative predictive values, and reaction time under a controlled environment. Controlled environment was achieved by use of dark room and chin rest, which resulted in uniform illuminance, fixed display-observer distance and fixed postures. We could also control target prevalence and level of difficulty such as the target size, target type and duration of presentation, by preparing background images from normal screening examinations, by inserting a cut-out lesion onto one of the background images, and by the use of computer programs widely used in cognitive psychology. This way, we could measure accuracy and reaction times of radiologists, and thanks to known target prevalence, sensitivity, specificity, positive and negative predictive values as well. In both non-board and board groups, radiologists had high sensitivity, specificity, accuracy positive predictive values and negative predictive values. Radiologists also presented fast reaction times of less than 1 second for all target types in both groups, when the maximum duration of image presentation was 1,000 ms.

There were no significant differences between board and non-board radiologists for sensitivity, specificity, positive and negative predictive values in all target types. Since this experiment intentionally used obvious targets, this result rather proves successful experiment, and is not surprising. For reaction time, board radiologists were significantly faster in answering for bulla. Bulla had weaker contrast to the background lung field compared to GGO and SN. Considering this fact, board radiologists might have become faster at finding targets that do not stand out by experience.

We should discuss limitations of this study. First of all, the task level might have been too easy to derive difference between board and non-board radiologists: accuracy of both groups were higher than 95% for all target types. For one reason, we presented only one 20 cm square CT image in the display field following traditional studies in cognitive psychology, because smaller image display makes interpretation and validation of the results difficult. Our option was to use a tile display, but the viewing distance of 70 cm was too far to observe 4 or 9 images in 20 cm square. For the second reason, the target was large enough to be obvious to all the participating radiologists. To determine the target size, we first investigated the smallest SN size that can surely be discerned from grouped vessels on one plane, and made the sizes of bulla and GGN close to it. In future studies, animations of some consecutive images with a target on one of them may be used to deal with those limitations: animation shortens duration of target presentation, and enables to follow the continuity of vessels in multiple planes. For another limitation, the accuracy of the response and response time reflect the participants’ detective ability, as well as the elements of neurological response of the participants.

## Conclusion

We developed a feasible experimental system for measurement of radiologists’ performance by visual search experiment. Board participants had no significant difference from non-board radiologists in terms of accuracy, sensitivity, specificity, positive and negative predictive values and reaction times for GGN and SN, but presented significantly faster reaction time for bulla.
